# Medical Association of Georgia

**Published:** 1878-03

**Authors:** 


MEDICAL ASSOCIATION OF GEORGIA.—As announced in the February number of this JOURNAL, the annual meeting of the Medical Association of Georgia will take place on the third Wednesday, 17th of April, prox.

The physicians of Atlanta, we believe, will show their appreciation of this useful institution, not only by a general attendance during its deliberations, but by extending a hearty welcome to members from other parts of the State. We know of nothing likely to mar the harmony and good feeling which should and generally does exist at these reunions of the profession in Georgia.

We have always felt a lively interest in the Association, having been connected with it from its inauguration in 1849, as the record shows, but one or two years before that time, according to our recollection of the date. About thirty years, then, except about six years during and immediately after the war, has the Association held annual meetings.

A yearly change in the place of meeting being thought necessary to the success of the Association, the migratory system was adopted early in its history. The permanent organization was had in the city of Macon, according to my recollection of the time, in 1847 or 1848, one or two years earlier than the record of membership indicates. I very well remember my admission to membership when the meeting was held in rear of and near the Floyd House, at which the venerable Dr. L. D. Ford, of Augusta, then in the prime of life, was elected President, and so far as I know, the first regularly elected presiding officer of the "Georgia Medical Society," as I think it was then called. Since that time, changes have been made not only in the name, but in the time of electing officers. Dr. Ford, the first President, was elected at the meeting at which he presided, and so of other succeeding meetings, perhaps. Now the President is elected at the close of a session, to be inaugurated at the opening of the succeeding annual meeting.

To Macon must be awarded the honor of a mother's fostering care, and to Dr. Ford, our cherished preceptor, the distinction of giving the institution paternal direction which has resulted in a successful career of usefulness.

